# Retrospective analysis of hip arthroplasty in patients younger than 50 years in a single center

**DOI:** 10.12669/pjms.40.11.9453

**Published:** 2024-12

**Authors:** Naeem Ullah, Babur Farid, Saleem Ullah, Syed Imran Bukhari

**Affiliations:** 1 Dr. Naeem Ullah, FCPS. Department of Trauma & Orthopedics Lady Reading Hospital, Peshawar, Pakistan; 2 Dr. Babur Farid, MBBS. Department of General Surgery Lady Reading Hospital, Peshawar, Pakistan; 3 Dr. Saleem Ullah, MBBS. Department of Trauma & Orthopedics Lady Reading Hospital, Peshawar, Pakistan; 4 Dr. Syed Imran Bukhari, FCPS. Department of Trauma & Orthopedics Lady Reading Hospital, Peshawar, Pakistan

**Keywords:** Arthroplasty, Avascular necrosis, Cementless

## Abstract

**Objective:**

To report on causes and treatment options of hip arthroplasty in younger population in a single center.

**Methods:**

Data was accessed retrospectively from hospital information system (HIS) and charts were reviewed. All patients younger than 50 years, who had hip arthroplasty for any indication during January 2020 to July 2023 time period at Lady Reading Hospital, Peshawar, were included in this study.

**Results:**

We identified 61 patients, comprising of 33 male and 28 female patients. Mean age was 36.98 ± 7.792 years. Non-cemented hip arthroplasty was performed in 88.5% of patients. Avascular necrosis (49.2%) was the leading indication for hip arthroplasty in younger patients, followed by neglected fracture neck of femur (24.6%), inflammatory arthritis (3.2%), and other causes (23%). Metal on highly cross-linked polyethylene was the commonest bearing surface.

**Conclusion:**

Non-cemented hip arthroplasty was the commonest treatment method in younger population in this study.

## INTRODUCTION

Hip arthroplasty, or hip replacement surgery, is commonly performed in older individuals with degenerative joint diseases. However, the epidemiological studies of hip arthroplasty in the population reveals a growing trend in younger individuals requiring this procedure.^1^,^2^ This shift can be attributed to factors such as increased sports-related injuries, obesity, improvements in techniques and biomaterials and the desire for an active lifestyle and regain full activity.^3^,^4^ The success of total hip arthroplasty (THA) in older patients has also contributed to this trend. However, there are specific considerations and risk factors for young patients undergoing hip arthroplasty. These include an increased risk of perioperative complications due to the complex congenital, developmental and post traumatic abnormalities and early implant failure in young and obese patients.^5^,^6^ Patient age at the time of implantation and diagnosis are important factors in determining THA survivorship. The bearing materials used in the implants can influence implant survival, with improved highly cross-linked polyethylene (HXLPE) as a bearing material having a lower wear rate.^7^,^8^ According to Bayliss et al, the lifetime risk of requiring revision surgery in patients who had total THA over the age of 70 years was about 5% with no difference between sexes. In patients younger than 70 years, however, the lifetime risk of revision increased for younger patients, up to 35% for men in their early 50s, with large differences seen between male and female patients (15% lower for women in same age group).^9^ Evans JT concluded that surgeons can expect THA to last 25 years in around 58% of patients.^10^

Understanding the epidemiology of hip arthroplasty in young individuals is crucial for healthcare professionals to provide appropriate care and develop preventive measures. Current literature does not specifically mention cutoff age to differentiate between young and old population, but quite a few articles have reported on 45,55 and 65 years as arbitrary cutoff.^7^,^8^ According to Cieremans D, the demand for THA in the United States is expected to increase 174% from 2005 to 2030.^11^ It is estimated that 572,000 THAs will be done annually with 28% of these in patients younger than 55 years.^12^ This proportion is slightly lower outside the United States, with rates of 11.9%, 6.4% and 13.2% reported by the Canadian, United Kingdom and Australian joint replacement registries, respectively.^13^-^15^

Patients in low-income countries have peculiar problems arising out of poor access to healthcare facilities and illiteracy. Majority are employed as daily wagers and for these patients, taking leave to seek proper care means foregoing on their daily wage. Their first contact is usually the traditional bone setter and quacks and when their methods do not work, they are referred to proper care which is usually late. Majority of these patients are nutritionally deficient and beset with multiple co-morbidities.

Our rationale is to document the demographics and various indications and procedures performed in the past in younger patients. We intend to report on the long-term outcomes in future publications. As life expectancy is lower in South Asia, we are reporting on hip arthroplasty performed in patients ≤ 50 years.

## METHODS

This retrospective chart review included all patients (≤50 years) who had hip replacement surgery between January 2020 and July 2023 at the orthopedic and trauma surgery department of Lady Reading Hospital, Peshawar, Pakistan. The patient’s medical records were retrospectively reviewed using electronic medical records in the Hospital information system (HIS). Revision procedures were excluded. All the patients were admitted from outpatient department and after anesthesia assessment, they were operated usually after two days of admission. Patients were mobilized on first post-operative day and were routinely discharged on second post operative day. We collected the details on demographics, disease leading to the hip arthroplasty and type of procedures.

### Ethical Approval:

The Institutional Review Board of Lady Reading Hospital approved the research (Ref. No. 989/LRH/MTI). This study was carried out following the Helsinki Declaration.

### Statistical Analysis:

The data were analyzed using SPSS version 25. The frequencies and proportions were presented as point estimates for categorical variables, while the mean ± SD was employed wherever necessary for quantitative variables.

## RESULTS

**Fig.1:**
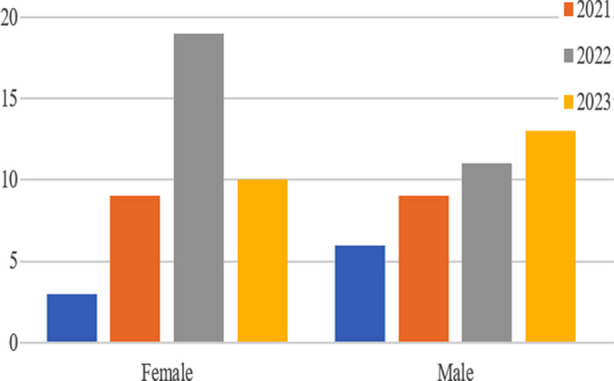
Number of patients stratified according to year and gender.

**Fig.2:**
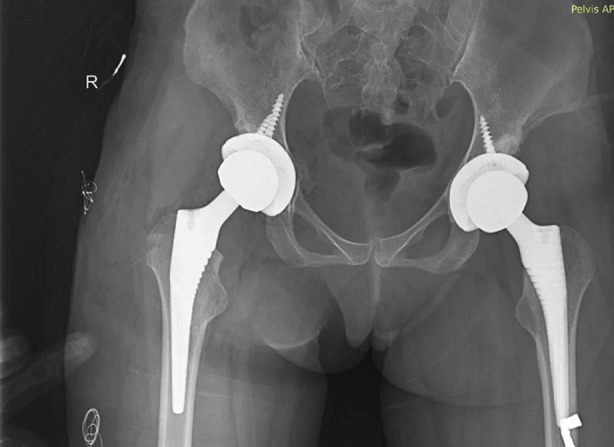
Bilateral Avascular necrosis in 23 year old female.

**Fig.3:**
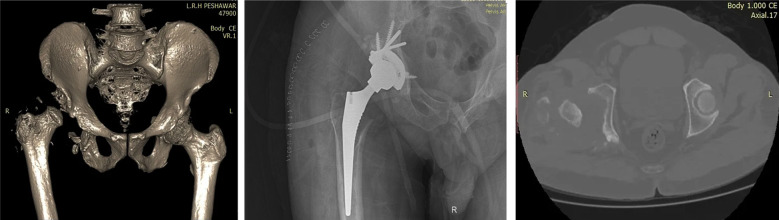
Post-traumatic Total hip replacement in 44 year old.

**Fig.4:**
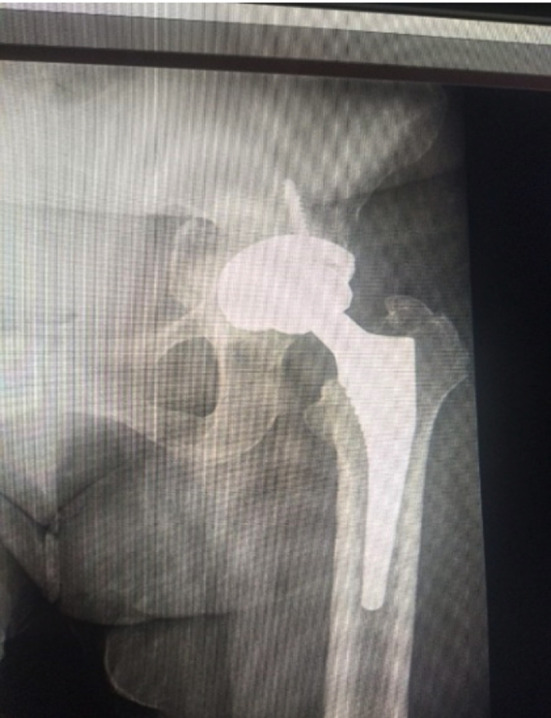
Total hip replacement in 32 year old patient with rheumatoid arthritis.

Sixty one patients were identified and included in this study (Fig.1). Gender distribution is shown in Table-I. Age distribution is shown in Table-II. Various procedures are outlined in Table-III. The procedures were done by different consultants of the department and implants of different manufacturers were used. Lateral (Hardinge) approach was used in all the procedures. Various diagnoses are outlined in Table-IV. Duration of injury in patients with neck of femur fracture before presenting to the hospital are shown in Table-V. Fractur neck of femur was identified in 15 patients, three patients had Garden Type-3 and 12 patients had Garden Type-4 fracture. Few of the cases are shown in Fig.2,3 and 4. In co-morbidities, one patient had hypertension, one patient had hypertension and diabetes and one patient had hypertension along with diabetes and ischemic heart disease.

**Table-I:** Gender Range.

	Frequency	Percent
Male	33	54.1
Female	28	45.9

Total	61	100.0

**Table-II:** Age Range.

Mean	36.98
Range	21-49
Std. Deviation	7.792

**Table-III:** The procedure involved calculating the percentages of non-cemented THR, cemented THR, cemented bipolar, and hybrid THR

	Frequency	Percent
Non-cemented THR		
Ceramic on Poly (6)	54	88.5
Metal on poly (48)		
Cemented THR	3	4.9
Cemented bipolar	1	1.6
Hybrid THR	3	4.9

Total	61	100.0

**Table-IV:** Diagnosis Legend.

	Frequency	Percent
Avascular necrosis	30	49.2
Neck of femur fracture	15	24.6
Inflammatory Arthritis	2	3.2
Secondary Arthritis	14	23.0
Sequelae of septic arthritis (3)		
Ankylosing spondylitis (3)		
Sequelae of Dysplasia (8)		

Total	61	100.0

**Table-V:** Time Since Neck of Femur Fracture.

Valid	15 patients
Mean	20.07 days
Median	7.00 days
Range	179 days
Minimum	3 days
Maximum	182 days

## DISCUSSION

The number of younger patients needing THA is increasing worldwide. Non-cemented THA is the treatment of choice for these patients. Our results show that the mean age in our study was 36.98±7.792. Kahlenberg CA in their study on young patients (≤ 35 years) had mean age of 25.4 years.^16^ The mean age in a systematic review on young patients (≤ 55 years) by Mei XY was 42±7.4 years.^7^ The mean age in a registry-based study in under 55 years cohort by Kumar A was 46.4 years.^17^ Our patients are relatively younger compared to other studies due to the fact that our inclusion criteria was ≤ 50 years while other studies have higher cut-off age limit.

The male to female ratio in our study was 54.1:45.9. In the systematic review by Mei XY, 47% were female.^7^ In the registry-based study by Kumar A, the ratio was 52 male vs. 48 female.^17^ In the study by Cieremans D, 51% were female.^11^ Our results are not different from previously published studies. The indications for surgery in our cohort were, avascular necrosis (AVN) 49.2%, fracture neck of femur (24.6%), secondary arthritis (23%) and inflammatory arthritis (3.2%). Kahlenberg CA reported the common diagnoses as AVN (30.6%), JIA (27.8%), developmental dysplasia of the hip (15.7%), and posttraumatic arthritis (11.1%)^16^. Mei XY in their systematic review showed that most common preoperative diagnoses were AVN (32.4%), primary or secondary arthritis (27.0%) and developmental dysplasia of the hip (19.5%).**7** Kumar A in their registry-based study showed predominant diagnoses for THA in young patients were osteoarthritis (82.6%), avascular necrosis (6%), inflammatory arthritis (2.4%), trauma (2.2%), and other causes (5.8%).^17^ Our results differ in some respect from other studies due to our peculiar socio-economic condition. Large proportion of our population have limited access to advanced healthcare facilities and they rely on quacks for first line treatment. Majority prescribe steroids and this may be one of the reasons that around half of our study population presented with AVN. Similarly, there is a large burden of neglected/mismanaged trauma due to poor access to healthcare facilities and as shown, mean duration of injury in those presenting with neck of femur fracture in our study was 20.07 days.

In our study, 88.5% patients had non-cemented THA, with ceramic-on-highly cross-linked poly bearing used in six patients and metal-on-highly cross-linked poly bearing used in 48 patients. In three patients, cemented THA was used; hybrid THA was used in three patients and cemented bipolar in one patient. In the systematic review by Mei XY in 2019, cementless fixation was used in 68.8%, hybrid in 16.8% and cemented in 14.4%. The bearing surface used was metal-on-conventional-polyethylene in 55.7%, ceramic-on-ceramic in 23.2%, ceramic-on conventional polyethylene in 16.5%, metal-on-highly cross-linked polyethylene in 4.6% and ceramic-on-highly-cross-linked polyethylene in 0.1%.^7^ In the study by Kumar A in 2017, the most common bearing couple used in THA was ceramic on ceramic, accounting for 66% of cases, followed by ceramic on polyethylene (15%).^17^ Zagra L in their review suggested that below the age of 60 years, cementless fixation with CoC bearing surface should be used.^18^ Tsikandylakis G recommended that the choice between metal on highly cross-linked poly, Ceramic on highly cross-linked poly and Ceramic on ceramic articulation could be expected to provide similar clinical results up to 10–15 years.^19^ We have used metal-on-highly cross-linked poly in majority of cases and not ceramic, because of the cost factor and poor access to health insurance.

Our literature search has not yielded meaningful literature on hip arthroplasty in younger population in Pakistan. Our study highlights the importance of further research in Pakistan regarding hip arthroplasty in younger population.

### Limitations:

This is retrospective data collection on hip arthroplasty in young population. Outcomes have not been assessed to inform decision making.

## CONCLUSION

Non-cemented Total Hip Arthroplasty was the commonest treatment option in younger population.

### Authors contribution:

**SIB** conceived and designed the study. Critical Review.

**BF and SU** did data collection. Critical Review

**NU** did literature search, Critical Review.

All authors have read the final version and are accountable for the integrity of the study.
